# Effectiveness of Physical Activity Intervention on ADHD Symptoms: A Systematic Review and Meta-Analysis

**DOI:** 10.3389/fpsyt.2021.706625

**Published:** 2021-10-26

**Authors:** Yongtao Xie, Xuping Gao, Yiling Song, Xiaotong Zhu, Mengge Chen, Li Yang, Yuanchun Ren

**Affiliations:** ^1^College of Physical Education and Sports, Beijing Normal University, Beijing, China; ^2^Department of Human Movement Science, Hebei Sports University, Shijiazhuang, China; ^3^Department of Child & Adolescent Psychiatry, National Clinical Research Center for Mental Disorders and NHC Key Laboratory of Mental Health (Peking University Sixth Hospital), Peking University Sixth Hospital (Institute of Mental Health), Beijing, China

**Keywords:** attention-deficit/hyperactivity disorder, physical activity, intervention, motor skill, effectiveness

## Abstract

**Objective:** To assess the effectiveness of physical activity (PA) intervention on attention-deficit/hyperactivity disorder (ADHD)-related symptoms.

**Method:** Studies that investigated PA intervention for ADHD-related symptoms were identified through searching PubMed, Web of Science, Cochrane Library, and Embase databases from inception through June 2021. Standardized mean difference (SMD) with 95% confidence interval (CI) was used to assess the effectiveness of PA intervention on improving ADHD-related symptoms. The meta-analyses were conducted using fixed-effect or random-effect models according to the heterogeneity of the studies.

**Results:** Nine before–after studies (232 participants) and 14 two-group control studies (162 participants/141 controls) were included in this meta-analysis. Combined results for before–after studies indicated significant improvements on all studied ADHD-related symptoms (inattention: SMD = 0.604, 95% CI: 0.374–0.834, *p* < 0.001; hyperactivity/impulsivity: SMD = 0.676, 95% CI: 0.401–0.950, *p* < 0.001; emotional problems: SMD = 0.416, 95% CI: 0.283–0.549, *p* < 0.001; behavioral problems: SMD = 0.347, 95% CI: 0.202–0.492, *p* < 0.001). Meta-analyses for two-group control studies further confirmed that PA intervention significantly improved the inattentive symptom (SMD = 0.715, 95% CI: 0.105, 1.325, *p* = 0.022). Subgroup analyses suggested significant beneficial effect on inattention symptoms in children. Moreover, closed motor skills were beneficial for hyperactive/impulsive problems (SMD = 0.671, *p* < 0.001), while open motor skills were beneficial for attention problems (SMD = 0.455, *p* = 0.049). When excluding studies with combined medication, the studies in unmedicated participants in before–after studies still showed significant results in all studied ADHD-related symptoms as in the overall analysis. Given the limited sample size, the best frequency and intensity of PA intervention need further investigation.

**Conclusion:** Our results suggested that PA intervention could possibly improve ADHD-related symptoms, especially inattention symptoms. Closed-skill and open-skill activities could be beneficial for hyperactivity/impulsivity and inattention symptoms, respectively. Further high-quality randomized clinical trials with large sample size are needed.

## Introduction

ADHD is one of the most prevalent mental disorders in children and usually lasts into adulthood, with a worldwide prevalence of about 5.9% (1). In addition to the “core symptoms” of inattention, impulsivity, and hyperactivity, children with ADHD also often combine cognitive and behavioral problems, which cause multidimensional difficulties for their academic, emotional, and social functions (2–7).

At present, the major treatments for ADHD are medication (8) and behavioral/psychological therapy (9). Stimulant medication helps patients reduce aberrant classroom behavior, improve on-task behavior, and relieve academic difficulties. Reduced risk-taking behavior and increased self-esteem were also observed in ADHD children under stimulant medication (10). However, stimulants could have short-term side effects, for instance, insomnia, appetite suppression, headache, stomachache, or slight elevations in heart rate, and blood pressure (10). As the other effective treatment for children with ADHD, behavior therapy might be difficult to adhere due to high financial cost and time consuming (11, 12). In summary, a viable, accessible, sustainable, and effective therapy for ADHD is needed.

Growing evidence shows that moderate physical activity (PA) can improve psychological health through enhancement of neurotransmitter systems and upregulation of the brain-derived neurotrophic factor (BDNF) and neurogenesis (13). As a potential compliant intervention for ADHD, PA may play a physiological role similar to stimulant medications by increasing dopamine and norepinephrine neurotransmitters, thereby alleviating the symptoms of ADHD (14). Moreover, PA is associated with increased level of 5-hydroxytryptamine (5-HT) and endogenous opioids which may further enhance mood and attention (15). A 6-week prospective trial indicated that PA group (13 ADHD children participating in a 90-min athletic activity twice per week) had greater improvements in ADHD Rating Scale scores compared to education group (15 ADHD children receiving 12 sessions of education on behavior control) (16). Another interventional study suggested that 8-week (40 min per session, twice per week) yoga exercise could improve the sustained attention in children with ADHD (17). A repeated-measure crossover trial conducted in 32 adult men with symptoms of ADHD found that a 20-min bout of moderate-intensity cycle exercise could reduce feelings of confusion, fatigue, and depression (18). However, the effectiveness of PA intervention on ADHD symptoms remained controversial, with several studies reporting negative findings (19, 20).

Motor skills are defined as physical activities involving a single or a group of movements performed with a high degree of precision and accuracy (21). Based on players' reaction to the external environment, motor skills of PA can be generally divided into open skills and closed skills (21). Open motor skills are performed in an environment that is unpredictable or in motion that requires performers to adapt their movements in response to dynamic properties of the environment (22), while closed motor skills are performed in highly consistent, stationary, and self-paced environment (e.g., swimming or track and field) (23–25). Due to the specificity of motor skill types, PA may be beneficial for different brain functions (23). Besides, PA may differentially influence individuals according to the development stage and may be especially beneficial for ADHD symptoms in early development (26).

Given that the effect of PA on ADHD symptoms is a major concern clinically, we conducted this systematic review and meta-analysis to establish the clinical effectiveness of PA on ADHD related symptoms across ages and environmental settings.

## Method

This study was conducted in accordance with the Preferred Reporting Items for Systematic Reviews and Meta-Analyses guideline (Supplementary Table 1) (27). The primary research question is to evaluate the effect of PA intervention on ADHD-related symptoms in single-arm or two-arm control clinical trials. The secondary questions include comparing effect among different age groups, using different motor skills, motor intensity, and frequency, as well as with or without drug treatment.

### Search Strategies

We searched for all kinds of ADHD-related research published in English from inception to June 10, 2021. Electronic databases were searched for PubMed, Web of Science, Cochrane Library, and Embase. The following search syntax was used to find relevant literature: (attention deficit disorder with hyperactivit^*^ OR attention-deficit hyperactivity disorder^*^ OR attention deficit disorder^*^ OR attention deficit disorder^*^ with hyperactivity OR attention deficit hyperactivity disorder^*^ OR attention deficit-hyperactivity disorder^*^ OR attention deficit disorder hyperactivity OR attention deficit hyperkinetic disorder OR ADHD OR adhd OR addh OR add OR attention deficit OR child attention deficit disorder OR inattenti^*^ OR attention problem^*^ OR hyperactiv^*^ OR hyperkinetic syndrome^*^ OR syndrome^*^ hyperkinetic OR hyperkinetic syndrome childhood OR hyperkinetic disorder OR hyperkinet^*^ OR overactive^*^ OR overactive child syndrome OR syndrome hyperkinetic OR hyperkinetic syndrome OR hyperactivity disorder OR hyperactive child syndrome OR childhood hyperkinetic syndrome) and (sport^*^ OR exercis^*^ OR locomotor activit^*^ OR physical^*^ therap^*^ OR physical^*^ activit^*^ OR motor activit^*^ OR soccer OR swim^*^ OR aquatic^*^ OR dive OR diving OR football OR pin pang OR ping-pong OR ping pang OR basketball OR cricket OR tennis OR rugby OR danc^*^ OR athletic^*^ OR martial art^*^ OR netball OR hockey OR gym^*^ OR horse rid^*^ OR horseback rid^*^ OR equestrian OR baseball OR yoga OR badminton OR taekwondo OR danc^*^ OR judo OR cycling). PA is defined as any body movement produced by contraction of skeletal muscle that substantially increases energy expenditure (28). We identified additional records through manual searching for the reference lists of included studies. Detailed information on the study search process is presented in Supplementary Material.

### Study Selection

Two independent reviewers evaluated all potential articles for inclusion. Disagreements were resolved by discussion among all coauthors. Inclusion criteria were as follows: (1) clinical trials investigated PA intervention; (2) studies examined the effect of PA intervention on ADHD symptoms as compared to the no-intervention condition (either control group, pretest measure, or both); (3) study participants had clinical diagnosis of ADHD, or ADHD-related symptoms; and (4) studies reported ADHD-related symptom scores. Studies were included regardless of medication status (medications for ADHD or any other treatment) or sex ratio. We excluded literatures for the reasons below: (1) studies had not reported the data as mean/SD or were short of data of pretest or posttest; (2) ADHD symptoms are not measured using scale symptom scores, (for example, reaction time); (3) study compared with other treatment group (medication group, or neurofeedback group). Comorbidities in all or part of the study participants were exclusionary.

### Data Extraction

The following data were extracted from each study: first author, publish year, study design, diagnostic criteria for ADHD, sample size, participant characteristics (age, sex, and race), PA programs (intensity based on the study description, duration of per session, frequency of PA intervention per week, and type of motor skills), and measurements of ADHD symptoms. If the ADHD symptom scores were only provided in figures, we extracted data from the bar charts using GetData Graph Digitizer v.2.25 software (http://getdata-graph-digitizer.com/) (29, 30).

### Effect Size

According to the Cochrane handbook for systematic reviews of interventions (31), all effect sizes were compared based on SMD, because eligible studies assessed the same ADHD symptoms using different psychometric scales. The ADHD-related symptoms were divided into four categories, including inattention, hyperactivity/impulsivity, emotional problems, and behavioral problems. The core symptoms of hyperactivity and impulsivity were generally assessed together (19, 32–40) and hence were considered as one outcome. A variety of emotional and behavioral problems were evaluated in eligible studies, but using different psychometric scales. Followed the previous study (41), we used integrated outcomes for emotional and behavioral problems; the items included in each integrated outcome are provided in Supplementary Table 2. To facilitate statistical independence of the data (42), for studies that used more than one version of scales (i.e., self, parent, and teacher version) to measure the same ADHD symptoms, we combined the effect sizes to guarantee that each study would contribute one effect size per outcome domain (i.e., inattention, hyperactivity–impulsivity, emotional and behavior problems).

### Statistical Analysis

In consideration of the heterogeneity of included studies and to control the impact of the self-healing factor, we conducted two separated meta-analyses for before–after studies (pre-intervention vs. post-intervention in one individual) and two-group control studies (PA intervention group vs. PA free group).

All statistical analyses were conducted in Stata software, version 14.0 (StataCorp LP, College Station, TX, USA). SMD with 95% confidence interval (CI) was calculated for each outcome. The heterogeneity of the included results was quantified by *I*^2^ and combined with the *p*-value to determine which model to be used. *I*^2^-values were interpreted as low (25%), moderate (50%), and high (75%) (43). Following recommendations by Field and Gillett (44), the random-effect model was used when *I*^2^ > 50% and *p* < 0.05; otherwise, the fixed-effect model was applied. Statistical significance was set at *p* < 0.05.

In order to find the source of heterogeneity, subgroup analyses were conducted stratified by ADHD diagnostic status (yes vs. no), study population (children vs. adults), type of motor skills (closed motor skills vs. open motor skills), PA intensity (moderate intensity vs. moderate-to-vigorous intensity), and PA frequency (<3 times per week vs. ≥3 times per week). We also analyzed the effect in the non-medicated group but were unable to compare un-medicated with medicated group due to limited study (only 1) for the latter. Publication bias was examined based on asymmetry inspection and quantified by Egger's linear regression method (45). Moreover, the potential publication bias was adjusted using non-parametric rank-based data augmentation techniques (trim-and-fill procedure) developed by Duval and Tweedie (46).

## Result

### Search Results

**Figure 1:**
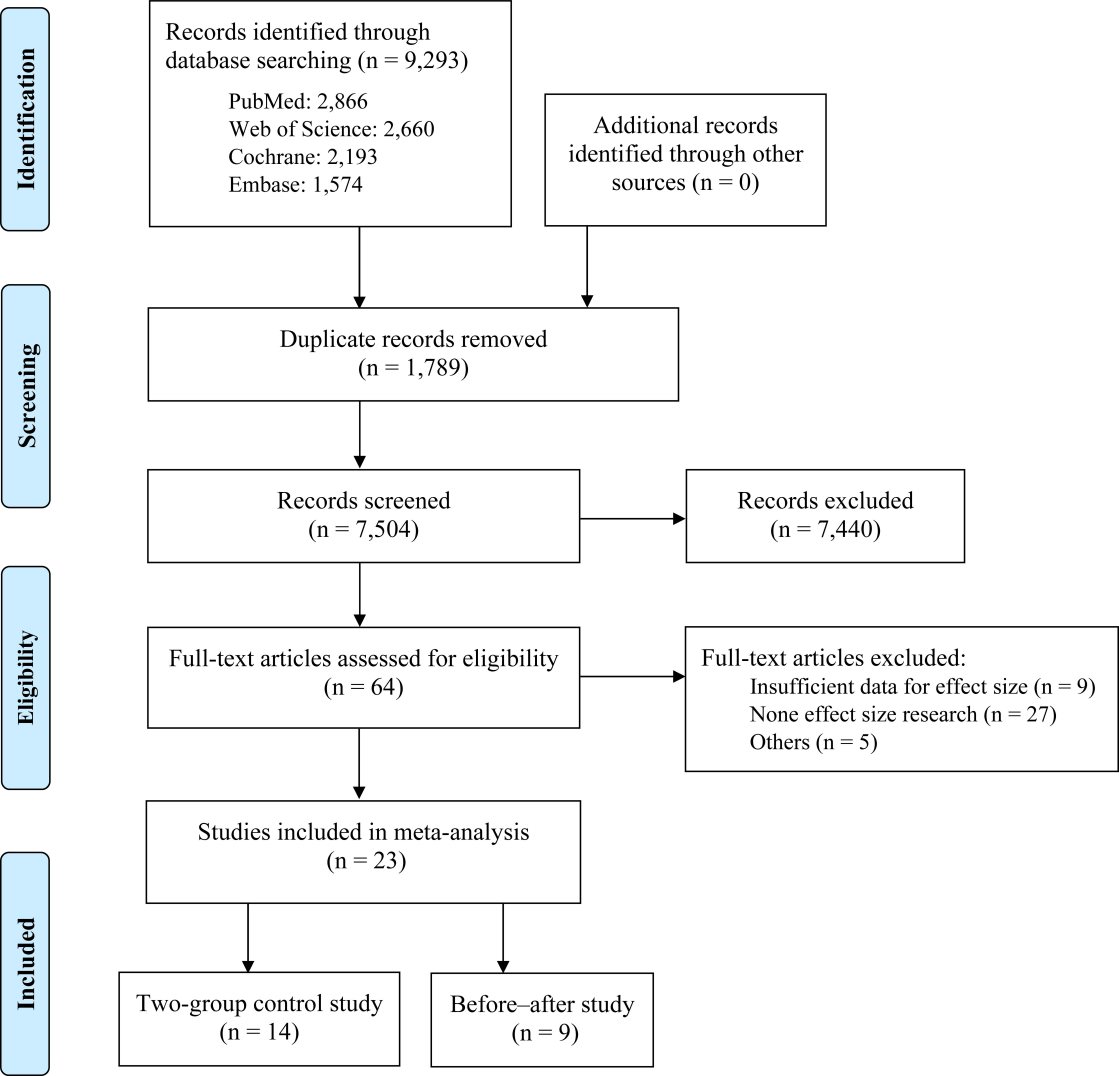
PRISMA flowchart.

A total of 9,293 articles were identified through searching four electronic databases, and 7,504 papers were left after removed duplicated literatures. By screening the abstract and the full text, 7,440 papers were further excluded due to non-English literature, no full text, or irrelevance. Finally, nine self-control trials (18, 37–40, 47–50), five non-randomized control trials (19, 32, 33, 51, 52), and nine randomized control trials (16, 20, 34–36, 53–56) were eligible for meta-analysis. The screening process is shown in Figure 1. The study design and demographic information of eligible studies is presented in Table 1, Supplementary Table 2. Most studies (20/23) were conducted among diagnosed ADHD participants and were mainly (20/23) focused on children and adolescents with age <18 years. Eleven studies did not provide the intensity information of PA intervention, and the remaining studies reported the effect of moderate (*n* = 5) or moderate-to-vigorous intensity (*n* = 7) PA intervention. During 4–24 weeks' PA intervention, the frequency of PA ranged from one to five times per week, and the duration ranged from 10 to 180 min each session. Stratified by the type of motor skills, 13 studies focused on open skills (e.g., horseback riding, racket-sports, and obstacle races), and 8 studies focused on closed skills (e.g., yoga, Tai Chi, and swimming). The following ADHD-related outcomes were reported across all included studies: inattention (*n* = 17), hyperactivity/impulsivity (*n* = 15), and emotional (*n* = 15) and behavioral problems (*n* = 12). Eight studies reported dropouts in the study samples. The average dropout rate was 20%.

**Table 1:** Summary of design and participant characteristics of studies included in meta-analyses.

**Study (year, country)**	**Study design**	**Diagnostic criteria**	**Medication**	**No. ^**a**^**	**Female (%)**	**Age (year)**	**Physical activity**				**Closed/open motor skills**	**Available outcomes**			
							**Intensity**	**Duration (min)**	**Frequency (times/week)**	**Week**		**Inattention**	**Hyperactivity/** **impulsivity**	**Emotional problems**	**Behavioral problems**
Jensen et al. (2004, Australia) (32)	Non-RCT	DSM-IV	NR	11/8	0	8–13	NR	NR	NR	NR	Closed	√	√	√	√
Verret et al. (2012, Canada) (19)	Non-RCT	DSM-IV	NR	9/9	10	7–12	Moderate-to-vigorous	45	3	10	Open	√	–	√	√
So et al. (2017, Korea) (51)	Non-RCT	Clinician	NR	10	NR	10–12	NR	30	2	4	Open	√	–	√	–
Kallweit et al. (2019, Germany) (52)	Non-RCT	DSM-IV	NR	36	44	22–41	Moderate-to-vigorous	10	NR	NR	Closed	√	√	–	—
Converse et al. (2020, America) (33)	Non-RCT	Clinician	NR	9/4	67	18–23	NR	60	2	7	Closed	√	√	–	—
Kang et al. (2011, Republic of Korea) (16)	RCT	Clinician	Yes	15/13	NR	7–9	Moderate	90	2	6	Closed	√	√	–	—
Hoza et al. (2015, America) (34)	RCT	DISC-IV	No	49/45	47	4–8	Moderate-to-vigorous	31	5	12	Open	√	√	√	√
Zhang et al. (2015, China) (53)	RCT	Clinician	NR	15/15	50	6–12	Moderate	30	3	10	Open	–	–	√	—
Pan et al. (2016, China) (55)	RCT	DSM-IV	NR	16/16	0	6–12	Moderate	70	2	12	Open	√	–	√	√
Bustamante et al. (2016, America) (54)	RCT	DISC-IV	NR	19/16	31	6–12	Moderate-to-vigorous	105	5	10	Open	√	√	–	√
García-Gómez et al. (2016, Spain) (20)	RCT	Clinician	NR	9/5	14	7–14	NR	45	2	12	Open	√	√	√	√
Geladé et al. (2017, Netherlands) (35)	RCT	DSM-IVTR	No	31	32	7–13	Moderate-to-vigorous	20	3	10	NR	√	√	–	–
Oh et al. (2018, Korea) (36)	RCT	DSM-IVTR	No	17	12	6–12	NR	60	2	12	Open	√	√	√	√
Silva et al. (2019, Brasilia) (56)	RCT	DSM-IV	NR	10/10	30	11–14	Moderate	45	2	8	Closed	–	–	√	–
Hernandez-Reif et al. (2000, America) (47)	Self-control	Clinician	NR	13	15	13–16	NR	NR	2	NR	Closed	–	√	√	√
Lufi (2011, Israel) (49)	Self-control	DSM-IVTR	No	15	0	8–13	NR	90	1	20	Open	√	–	√	√
Smith et al. (2013, America) (50)	Self-control	DSM-IV	No	14	12	5–8	Moderate-to-vigorous	26	5	8	Open	–	√	–	√
Cuypers et al. (2011, Norway) (48)	Self-control	Clinician	Yes	5	0	10–11	NR	60	2	24	Open	–	–	√	√
Fritz et al. (2015, America) (18)	Self-control	ASRS V1.1	No	32	0	18–34	Moderate	20	NR	NR	Closed	–	–	√	–
Jang et al. (2015, Republic of Korea) (37)	Self-control	DSM-IVTR	No	20	5	6–13	NR	30–60	2	12	Open	√	√	–	–
Schoenfelder et al. (2017, America) (38)	Self-control	Clinician	NR	11	54	14–18	NR	NR	NR	NR	Closed	√	√	√	–
Shema-Shiratzky et al. (2019, Israel) (39)	Self-control	DSM-5	No	14	21	8–11	NR	90–180	3	6	Open	√	√	√	√
Siu et al. (2020, China) (40)	Self-control	Clinician	NR	14	29	7–9	Moderate-to-vigorous	90	1	6	Open	√	√	–	–

a *The sample size of each study, the number before slash indicated the intervention group size, and the number after the slash indicated the control group size*.

*NR, not report; RCT, randomized control trial*.

### Overall Effects

**Figure 2:**
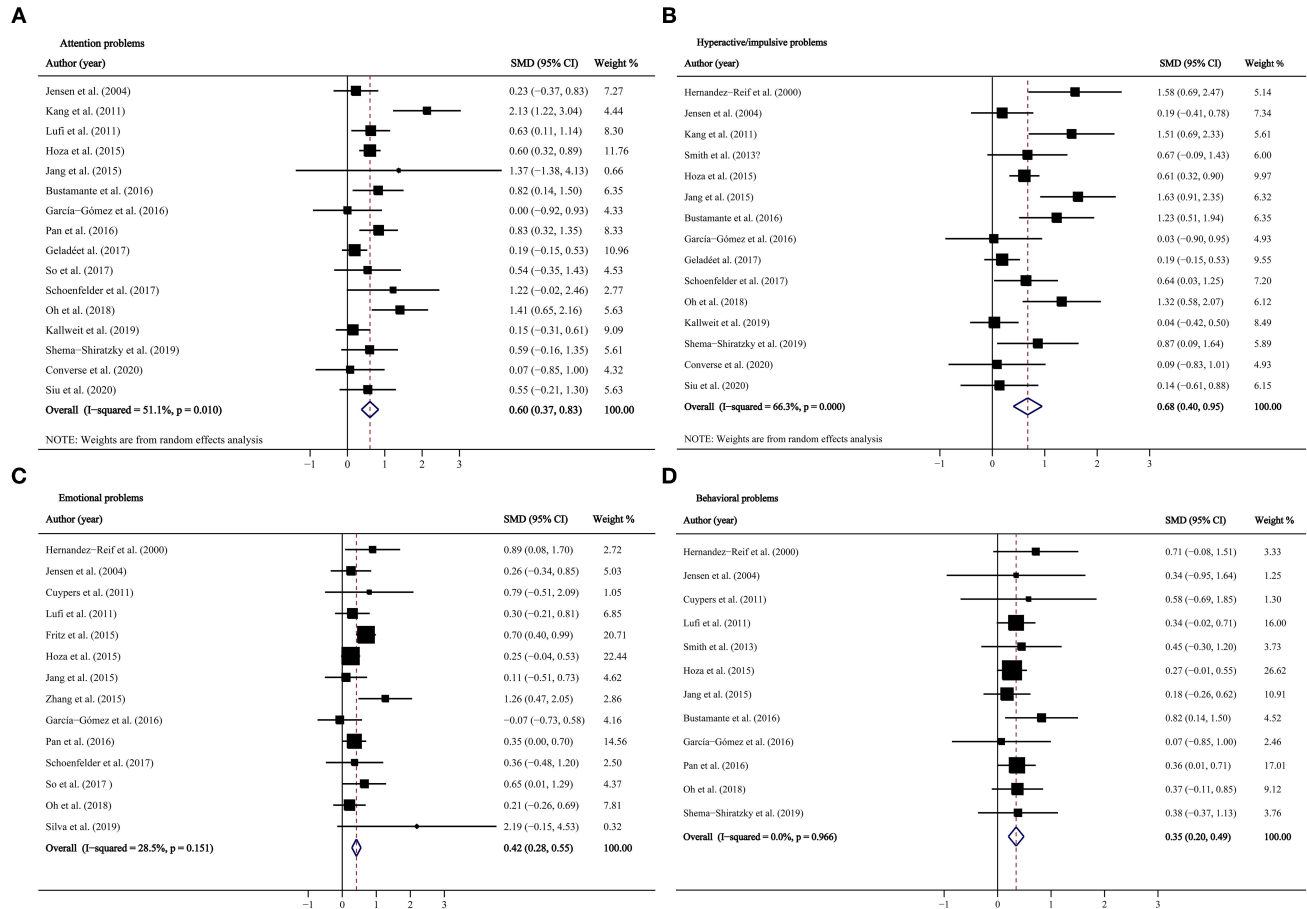
Forest plots for physical activity intervention on ADHD symptoms in before–after studies. **(A)** Attention problems; **(B)** hyperactive/impulsive problems; **(C)** emotional problems; **(D)** behavioral problems. CI, confidence interval; SMD, standardized mean difference.

**Figure 3:**
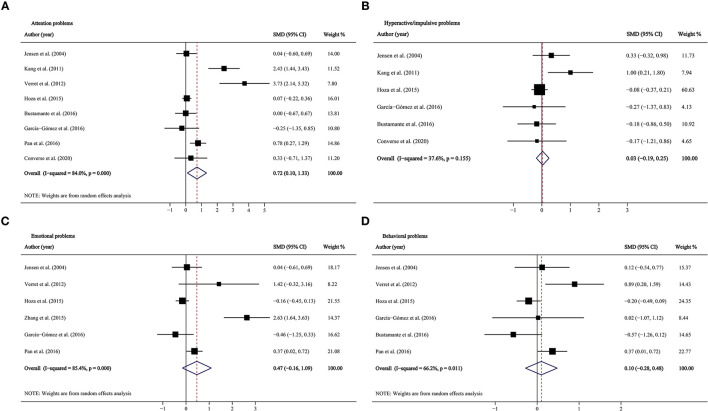
Forest plots for physical activity intervention on ADHD symptoms in two-group control studies. **(A)** Attention problems; **(B)** hyperactive/impulsive problems; **(C)** emotional problems; **(D)** behavioral problems. CI, confidence interval; SMD, standardized mean difference.

Combining the effect sizes from before–after studies, we detected significant improvements in all ADHD-related symptoms (inattention: SMD = 0.604, 95% CI: 0.374–0.834, *p* < 0.001; hyperactivity/impulsivity: SMD = 0.676, 95% CI: 0.401–0.950, *p* < 0.001; emotional problems: SMD = 0.416, 95% CI: 0.283–0.549, *p* < 0.001; behavioral problems: SMD = 0.347, 95% CI: 0.202–0.492, *p* < 0.001; Figures 2, 3), but heterogeneity was observed in pooled estimates for inattention and hyperactivity/impulsivity (*I*^2^ were 51.1 and 66.3%, respectively). The combined effect sizes of the two-group control studies suggested that PA intervention only significantly improved the attention problems (SMD = 0.715, 95% CI: 0.105, 1.325, *p* = 0.022, *I*^2^ = 84%; Table 2; Figure 3), whereas there was no significant effect on hyperactive/impulsive, emotional, and behavioral problems (*p*-values ranged from 0.141 to 0.787; Figure 3).

**Table 2:** Pooled effect size for physical activity intervention on attention problems.

	**Before–after studies**						**Two-group control studies**						
	**No**.	**Effect size**			**Heterogeneity**		**No**.	**Effect size**				**Heterogeneity**	
		**SMD**	**95% CI**	**P for Z**	***I* ^**2**^**	***P***		**SMD**	**95% CI**	**P for Z**		***I* ^**2**^**	***p***
**Overall intervention effect**	16	0.604	0.374, 0.834	<0.001	51.1%	0.010	8	0.715	0.105, 1.325	0.022		84.0%	<0.001
**ADHD diagnostic status**													
Yes	14	0.603	0.320, 0.885	<0.001	56.1%	0.005	6	1.056	0.140, 1.972	0.024		84.8%	<0.001
No	2	0.635	0.371, 0.899	<0.001	0.0%	0.560	2	0.059	−0.204, 0.323	0.659		0.0%	0.851
**Study population**													
Children	14	0.678	0.431, 0.926	<0.001	50.8%	0.015	7	0.776	0.101, 1.451	0.024		86.3%	<0.001
Adults	2	0.135	−0.278, 0.549	0.522	0.0%	0.881	1	0.327	−0.714, 1.368	0.538		–	–
**Motor skills**													
Closed	5	0.687	−0.020, 1.395	0.057	77.0%	0.002	3	0.909	−0.540, 2.359	0.219		87.6%	<0.001
Open	10	0.671	0.487, 0.855	<0.001	0.0%	0.641	5	0.604	−0.127, 1.336	0.105		84.3%	<0.001
**Intensity of physical activity**													
Moderate	2	1.426	0.163, 2.689	0.027	83.0%	0.015	2	1.550	−0.066, 3.166	0.060		88.1%	0.004
Moderate-to-vigorous	5	0.419	0.175, 0.663	0.001	33.6%	0.197	3	0.951	−0.328, 2.230	0.145		90.0%	<0.001
**Frequency of physical activity**													
<3 times per week	9	0.787	0.392, 1.182	<0.001	53.8%	0.027	4	0.838	−0.133, 1.808	0.091		79.5%	0.002
≥3 times per week	4	0.488	0.215, 0.760	<0.001	33.6%	0.210	3	0.951	−0.328, 2.230	0.145		90.0%	<0.001
**Medication**													
Yes	1	1.512	0.694, 2.330	0.000	–	–	–	–	–	–	–	–	–
No	6	0.993	0.349, 1.636	0.002	83.8%	0.000	–	–	–	–	–	–	–

*95% CI, 95% confidence interval; SMD, standardized mean difference*.

### Subgroup Analyses

Subgroup analyses were conducted according to ADHD diagnostic status. Combined results of before–after studies showed that PA intervention was effective for all studied symptoms, which were not different among diagnosed and suspected ADHD cases (P for interaction ranged from 0.599 to 0.871, Tables 2, 3, Supplementary Tables 3, 4). Based on two-group control studies, PA intervention was more effective for inattention and behavioral problems among diagnosed ADHD patients (P for interaction were 0.040 and 0.001, Table 2, Supplementary Table 4).

**Table 3:** Pooled effect size for physical activity intervention on hyperactive/impulsive problems.

	**Before–after studies**						**Two-group control studies**						
	**No**.	**Effect size**			**Heterogeneity**		**No**.	**Effect size**				**Heterogeneity**	
		**SMD**	**95% CI**	**P for Z**	***I* ^**2**^**	***P***		**SMD**	**95% CI**	**P for Z**		***I* ^**2**^**	***P***
**Overall intervention effect**	15	0.676	0.401, 0.950	<0.001	66.3%	<0.001	6	0.031	−0.192, 0.254	0.787		37.6%	0.155
**ADHD diagnostic status**													
Yes	13	0.650	0.322, 0.978	<0.001	68.2%	<0.001	4	0.350	−0.068, 0.768	0.101		37.8%	0.185
No	2	0.828	0.251, 1.405	0.005	59.2%	0.117	2	−0.096	−0.360, 0.168	0.475		0.0%	0.791
**Study population**													
Children	13	0.770	0.478, 1.062	<0.001	65.3%	0.001	5	0.041	−0.188, 0.269	0.727		49.1%	0.097
Adults	2	0.049	−0.364, 0.463	0.815	0.0%	0.921	1	−0.172	−1.207, 0.863	0.745		–	–
**Motor skills**													
Closed	6	0.624	0.106, 1.143	0.018	71.2%	0.004	3	0.455	0.002, 0.907	0.049		41.1%	0.183
Open	8	0.818	0.466, 1.170	<0.001	55.6%	0.027	3	−0.105	−0.362, 0.151	0.420		0.0%	0.924
**Intensity of physical activity**													
Moderate	1	1.512	0.694, 2.330	<0.001	–	–	1	1.004	0.213, 1.796	0.013		-	-
Moderate-to-vigorous	6	0.434	0.123, 0.744	0.006	58.3%	0.035	2	−0.096	−0.360, 0.168	0.475		0.0%	0.791
**Frequency of physical activity**													
<3 times per week	7	0.918	0.356, 1.480	0.001	69.9%	0.003	3	0.254	−0.613, 1.122	0.565		58.5%	0.090
≥3 times per week	5	0.617	0.283, 0.951	<0.001	54.1%	0.069	2	−0.096	−0.360, 0.168	0.475		0.0%	0.791
**Medication**													
Yes	1	2.129	1.222, 3.037	0.000	–	–	–	–	–	–	–	–	–
No	5	0.837	0.339, 1.335	0.001	71.0%	0.008	–	–	–	–	–	–	–

*95% CI, 95% confidence interval; SMD, standardized mean difference*.

As the development progresses, the plasticity of brain decreases and exercise could be an effective way to improve brain plasticity in children and adolescent (57, 58). We conducted subgroup analyses stratified by children/adolescent and adults. Pooled effect sizes for inattention indicated significant effect in both before–after studies and two-group control studies for children (before–after studies, SMD: 0.678, *p* < 0.001; two-group control studies, SMD: 0.776, *p* = 0.024, Table 2). However, the heterogeneities were high, suggesting that the results could be unstable. There were not enough studies (currently only three) for adults to get significant findings.

Stratified by the type of motor skills, the intervention effects of open skills and closed skills on ADHD symptoms were inconsistent. Results from before–after studies, only open motor skills intervention showed a significantly positive effect on inattention (SMD = 0.671, *p* < 0.001, Table 2), and effects of closed and open motor skills intervention had no difference on hyperactive/impulsive, emotional, and behavioral problems (P for interaction ranged from 0.201 to 0.544, Table 3, Supplementary Tables 3, 4). In two-group control studies, the pooled results indicated that closed skills could improve the hyperactive/impulsive problems (SMD = 0.455, *p* = 0.049), but open skills could not (P for interaction = 0.035, Table 3).

Subgroup analyses were also conducted stratified by the intensity and frequency of PA intervention. Compared to moderate-to-vigorous PA, moderate intervention showed better effect on hyperactive/impulsive problems (before–after study, SMD: 1.512 vs. 0.434, P for interaction = 0.016; two-group control, SMD: 1.004 vs. −0.096, P for interaction = 0.010, Table 3). Moreover, pooled effect size of before–after studies indicated that only lower frequency of PA intervention had significant effect on improving emotional problems (*P* for *Z*-test was 0.001 for <3 times per week, and 0.175 for ≥3 times per week, Supplementary Table 4).

In the un-medicated group, combined effect sizes for all ADHD-related symptoms showed consistent results as in overall analyses (inattention, SMD = 0.993, 95% CI: 0.349–1.636, *p* = 0.002; hyperactivity/impulsivity: SMD = 0.837, 95% CI: 0.339–1.335, *p* = 0.001; emotional problems: SMD = 0.268, 95% CI: 0.021–0.516, *p* = 0.033; behavioral problems: SMD = 0.332, 95% CI: 0.086–0.578, *p* = 0.008; Tables 2, 3, Supplementary Tables 3, 4).

### Publication Bias

Egger's tests revealed no significant publication bias, and the *p*-values ranged from 0.137 to 0.720. The funnel plots also showed no evidence of publication bias (Supplementary Figure 1).

## Discussion

ADHD is characterized by core symptoms of inappropriate levels of inattention, hyperactivity, and impulsivity. Moreover, the emotional and behavioral problems of patients are also prominent in ADHD, for instance, depression, oppositional defiant disorder, etc. In this systematic review and meta-analysis, we first assessed the effectiveness of PA intervention on ADHD symptoms. Combined estimates for before–after studies suggest significant improvements on all studied ADHD symptoms (i.e., inattention, hyperactivity–impulsivity, emotional and behavior problems). While meta-analyses for two-group control studies confirm that PA intervention can significantly improve the attention problems in ADHD. PA may be especially beneficial for inattention and hyperactivity/impulsivity symptoms in children/adolescents compared to adults. Subgroup analysis stratified by ADHD diagnostic status indicated that compared with subthreshold ADHD participants, the improvement effect of PA intervention on ADHD patients was more significant. Closed motor skills are beneficial for hyperactive/impulsive problems, and open motor skills are beneficial for inattention problems. However, we did not find the evidence that different intensity and frequency of PA can lead to different effects. PA intervention on un-medicated patients also had significant effect on ADHD symptoms.

Increasing evidence shows that PA plays a role in improving psychological health. A meta-analysis including 13 trials suggested a short-term effect of exercise on depression (59). Frederiksen et al. reviewed eight articles and suggested PA add-on was more beneficial for anxiety populations compared to cognitive behavioral therapy only (60). Recent systematic review by Ng et al. indicated that PA is a beneficial and well-tolerated intervention for children and adolescents with ADHD (14). Moreover, propriate sports context was suggested to be beneficial for social skills generalization among ADHD children (61). Although there were some studies with negative results (20, 32), our meta-analysis suggested that PA can be effective. The difference in results might be due to the heterogeneity of experimental methods, including the intervention methods and experimental design (62), which we tried to address in the following subgroup analyses. In consideration of some inconsistent results in subgroup analyses, the overall effect should be careful interpreting about the effects of PA on ADHD.

The exact mechanisms by which PA intervention is beneficial for inattentive symptom in ADHD patients are still unknown. Currently, the leading biological hypothesis of ADHD is based on the dysfunction of catecholamines (CAs, e.g., norepinephrine, epinephrine, or dopamine) (63). As one of the pathophysiology factors for ADHD, dopamine dysregulation in the brain dopamine reward pathway was suggested to be associated with inattentive symptoms in patients with ADHD, but not with hyperactive symptoms (64, 65). The CA response to environmental stimuli is attenuated in ADHD; therefore, methylphenidate and amphetamine (CA agonists) are effective in treating the symptoms of ADHD (66). Studies have reported that exercises might augment the synthesis and releases of dopamine and other CA in the prefrontal cortex, nucleus accumbens, caudate nucleus, and basal ganglia (67–69). Based on the above results, the underlying mechanism for PA intervention improving inattentive symptoms may be mainly associated with increased dopamine levels in the brain.

Subtype analysis in the children/adolescent group found significant improvement of ADHD inattention symptoms. Although the results might be biased by the high heterogeneity, it provided further evidence in young patients. A meta-analysis indicated that the efficacy of non-pharmacological interventions can vary substantially between children/adolescents and adults with ADHD (70). Some animal studies showed that the capacity of exercise-induced neuroplasticity decreases with age increase, so early brain rehabilitation in young children might play a key role in the treatment of ADHD symptoms (57, 71). On the other hand, a systematic literature review suggested that physical exercise also represented an effective treatment in adults with ADHD with regard to behavioral and socioemotional functions (72). In this study, the improvement in adults was not significant, which might be limited by the number of literatures.

There are various forms of PA intervention for ADHD, including walking, treadmill, and yoga (73–75). Stratified by the type of motor skills, our results indicated that closed motor skills are beneficial for hyperactive/impulsive problems, while open motor skills are beneficial for attention problems. The main difference between open and closed motor skills was suggested to be environment within which the activity is performed (23). Compared to closed-skill sports, open-skill sports associate with changing environment and diversity of the responses under high time pressure which demand a higher level of executive control in advance anticipation (i.e., online updating of the external changes and predicting the related outcomes) (76, 77) and imperative responses (i.e., initiating in-time actions in response to the external changes, including initiation of appropriate actions and inhibition of inappropriate actions) (78, 79). Therefore, open-skill sports require more visual attention and fast and flexible decision making and action execution (79), which makes open-skill sports more beneficial for inattentive symptom. In contrast, closed-skill sports make individuals tend to follow set patterns and hence be more consistent (80). Given the basically stable and high predictable external environment, closed-skill sports practitioners mainly rely on proprioception sensory feedback to adjust the movement. Based on these characteristics, it was not difficult to understand that closed-skill sports were more beneficial for hyperactive/impulsive problems.

Potential dose response may influence the effectiveness of PA intervention on ADHD symptoms. Subgroup analyses suggested that moderate intensity of PA intervention might have better performance, which was inconsistent with the finding by Ng et al. (14) that moderate-to-intense aerobic exercise might more beneficial for children and adolescents with ADHD. Moreover, study by Piepmeier et al. (81) indicated an inverted U-shaped relationship between exercise intensity and cognitive improvement. Propriate PA intervention intensity might associate with better attention allocation, higher information processing speed, and optimal physiological arousal after exercise (81). Our study highlighted that the dose of PA can be a key factor for ADHD intervention. However, due to the incomplete information and different settings of eligible studies, our results should be interpreted with caution, and more studies are needed to define the best intensity and frequency of PA intervention.

Although PA is beneficial for ADHD, whether PA can be used as an effective treatment is still controversial. A study argued that PA is only an adjunctive treatment for ADHD (82). Different from that study, our results that came from subgroup analyses in unmedicated patients suggested that PA may be an effective stand-alone treatment. This is because single PA intervention might improve the core symptoms and emotional and behavioral problems of ADHD.

The present systematic review and meta-analysis included a relatively large number of clinical trials allowing the preliminary exploration on effectiveness of PA intervention on ADHD symptoms. Nevertheless, there were also several limitations in this study. First, the limited number of participants might have resulted in a low statistical power to detect underlying differences. Although 23 eligible studies were included, the insignificant findings should be interpreted with caution. To assess the effectiveness of PA intervention, more high-quality clinical trials with large sample are needed. Second, individual studies might account for the findings in subgroup analyses. Given the insufficient statistical power for small sample size, subgroup analyses are exploratory, and these findings need further confirmation. Third, the measurements of emotional and behavior problems varied across studies, which might introduce confounders to the pooled estimates. However, the *I*^2^-values suggested no significant heterogeneity in the pooled effect size for emotional and behavior problems, and *I*^2^-values were 28.5 and 0.0%, respectively. Fourth, most study participants in this analysis were children; hence, extrapolating these findings to adult populations should be taken with caution. Finally, the effect of PA intervention can be impacted by other factors that may be not fully considered in the present study, including biological factors (e.g., age, gender), environmental factors (e.g., diet, sleep quality, other medication use), and intervention settings (e.g., the dose and intensity of PA). Future studies are needed to provide more information about these factors.

## Conclusion

Our results suggest that PA intervention could possibly improve ADHD-related symptoms, especially inattention symptoms. However, due to a lot of confounders, such as age, gender, ADHD subtypes, the lack of rigorous double-blinded randomized-control studies, and the inconsistency of the PA program, our results still need to be interpreted with cautions. Whether PA could be an effective alternative therapy to improve the clinical symptoms of ADHD and how to provide targeted exercise intervention for patients with different ages, subtypes, symptoms, and functional impairments need more accumulation of relevant evidence to be confirmed.

## Data Availability Statement

The original contributions presented in the study are included in the article/Supplementary Material, further inquiries can be directed to the corresponding author/s.

## Author Contributions

YR: took responsibility for the integrity of the data and the accuracy of the data analysis. YR and LY: study concept and design, MS editing, and study supervision. YX, XG, YS, XZ, and MC: data extraction and analysis. YX and XG: drafting of the manuscript. All authors reviewed and revised the article, gave final approval of the version to be published, and agree to be accountable for all aspects of the work.

## Funding

This work was supported by grants from National Key R&D Program of China (2016YFC1306103), the Major State Basic Research Development Program of China (973 Program, 2014CB846100), National Natural Science Foundation of China (81761128035), and the Ministry of Education of Humanities and Social Science Foundation (21YJA890025).

## Conflict of Interest

The authors declare that the research was conducted in the absence of any commercial or financial relationships that could be construed as a potential conflict of interest.

## Publisher's Note

All claims expressed in this article are solely those of the authors and do not necessarily represent those of their affiliated organizations, or those of the publisher, the editors and the reviewers. Any product that may be evaluated in this article, or claim that may be made by its manufacturer, is not guaranteed or endorsed by the publisher.
