# Corrigendum: Effectiveness of Physical Activity Intervention on ADHD Symptoms: A Systematic Review and Meta-Analysis

**DOI:** 10.3389/fpsyt.2021.806241

**Published:** 2021-12-06

**Authors:** Yongtao Xie, Xuping Gao, Yiling Song, Xiaotong Zhu, Mengge Chen, Li Yang, Yuanchun Ren

**Affiliations:** ^1^College of Physical Education and Sports, Beijing Normal University, Beijing, China; ^2^Department of Human Movement Science, Hebei Sports University, Shijiazhuang, China; ^3^Department of Child & Adolescent Psychiatry, National Clinical Research Center for Mental Disorders and NHC Key Laboratory of Mental Health (Peking University Sixth Hospital), Peking University Sixth Hospital (Institute of Mental Health), Beijing, China

**Keywords:** attention-deficit/hyperactivity disorder, physical activity, intervention, motor skill, effectiveness

In the published article, there was an error in the order of the authors in the correspondence section. Yuanchun Ren should be listed before Li Yang.

In the original article, 3rd sentence of the 2nd paragraph in discussion section should be changed from “Frederiksen et al. reviewed eight articles and suggested that PA was more beneficial for anxiety populations compared to cognitive behavioral therapy (60).” to “Frederiksen et al. reviewed eight articles and suggested PA add-on was more beneficial for anxiety populations compared to cognitive behavioral therapy only (60).”

The authors apologize for this error and state that this does not change the scientific conclusions of the article in any way. The original article has been updated.

## Publisher's Note

All claims expressed in this article are solely those of the authors and do not necessarily represent those of their affiliated organizations, or those of the publisher, the editors and the reviewers. Any product that may be evaluated in this article, or claim that may be made by its manufacturer, is not guaranteed or endorsed by the publisher.
