# Corrigendum: Effectiveness of ertapenem for treatment of infections in children: An evidence mapping and meta-analysis

**DOI:** 10.3389/fped.2022.1095727

**Published:** 2023-01-09

**Authors:** Ruiqiu Zhao, Xiaoru Long, Jiangxia Wang, Jing Zhu, Cong Liu, Tingting Shang, Zhenzhen Zhang, Engels Obi, Lynda Osadebe, Yue Kang, Jie Liu, Xiaodi Chen, Hongmei Xu

**Affiliations:** ^1^ Chongqing Key Laboratory of Child Infection and Immunity, Chongqing Key Laboratory of Pediatrics, Ministry of Education Key Laboratory of Child Development and Disorders, Department of Infectious Diseases of Children’s Hospital of Chongqing Medical University, National Clinical Research Center for Child Health and Disorders, China International Science and Technology Cooperation Base of Child Development and Critical Disorders, Chongqing, China; ^2^ Merck & Co., Inc., Rahway, NJ, United States; ^3^ MRL Global Medical Affairs, MSD China, Shanghai, China

**Keywords:** children, infection, ertapenem, efficacy, safety

A Corrigendum on Effectiveness of ertapenem for treatment of infections in children: An Evidence mapping and meta-analysis

In the published article, there was an error in Figure 3, the “Favours” axis was inverted in the “Treatmenet success rate” subgroup. The corrected Figure 3 appears below.

**Figure 3:**
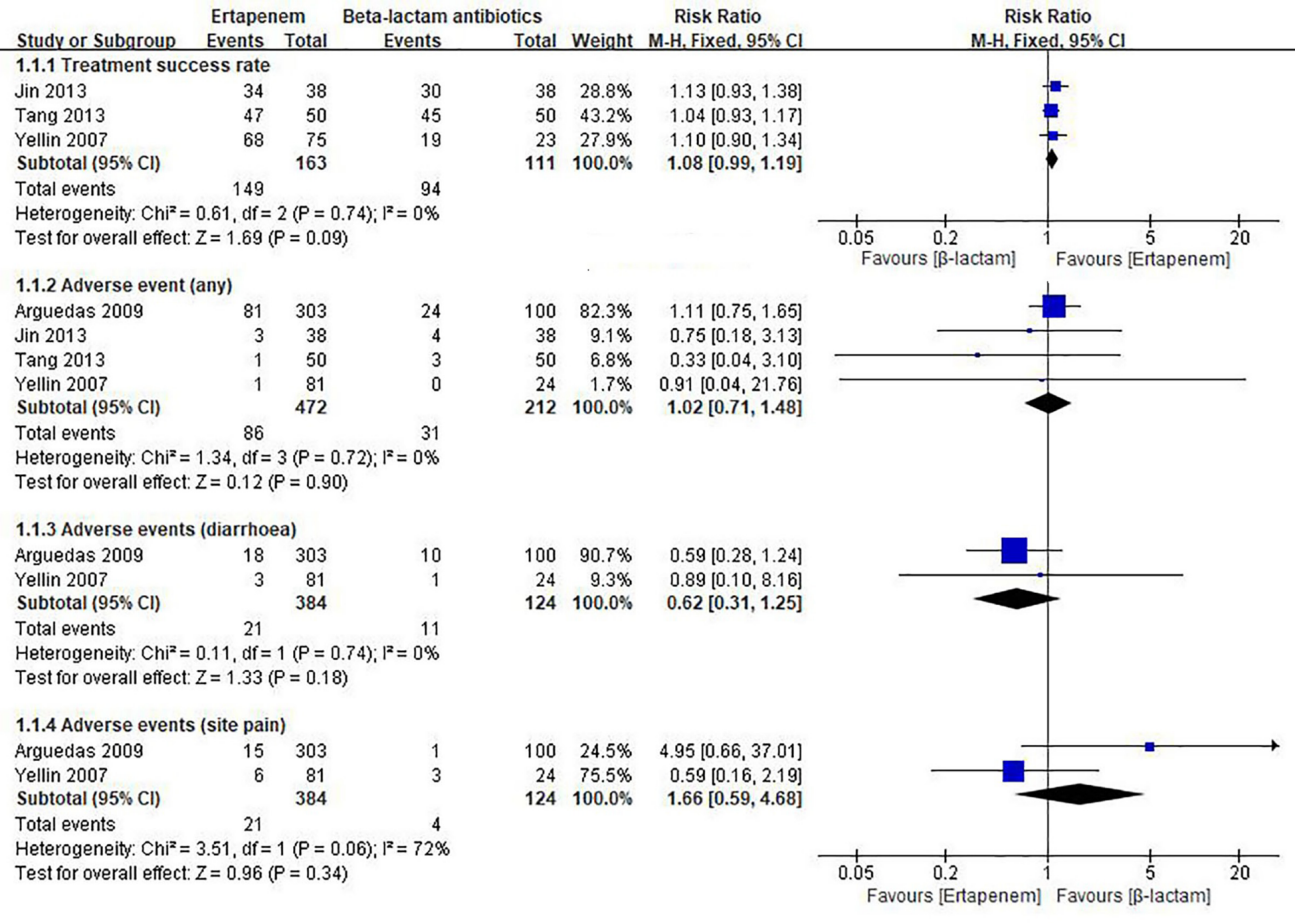
Meta-analysis for ertapenem vs. β-lactam antibiotics in children.

The authors apologize for this error and state that this does not change the scientific conclusions of the article in any way. The original article has been updated.

