# Supplementary material for: Effectiveness of Physical Activity Intervention on ADHD Symptoms: A Systematic Review and Meta-Analysis
Source: Front Psychiatry. 2021 Oct 26;12:706625. doi: 10.3389/fpsyt.2021.706625 (PMC8575983; doi:10.3389/fpsyt.2021.706625)
Supplement: Supplementary file 1 [file Data_Sheet_1.docx]

**Title Page**

**Effectiveness of Physical Activity Intervention on ADHD Symptoms: A Systematic Review and Meta-analysis**

**Yongtao Xie ^1, 2, †^, Xuping Gao ^3, †^, Yiling Song ^1^, Xiaotong Zhu ^1^, Mengge Chen ^1^, Li Yang ^3, *^, Yuanchun Ren ^1, *^**

^1^ College of Physical Education and Sports, Beijing Normal University, No.19 Xinjiekouwai Street, Beijing, 100875, PR China.

^2^ Department of Human Movement Science, Hebei Sports University, No.82 Xuefu Road, Shijiazhuang, Hebei, 050041, PR China.

^3^ Department of Child & Adolescent Psychiatry, Peking University Sixth Hospital (Institute of Mental Health), National Clinical Research Center for Mental Disorders and NHC Key Laboratory of Mental Health (Peking University Sixth Hospital), 51 HuayuanBei Road, Beijing, 100191, PR China.

^†^ Yongtao Xie and Xuping Gao contributed equally to this work and share co-first authorship.

**^*^ Correspondence:**

**Yuanchun Ren**, PhD

College of Physical Education and Sports, Beijing Normal University, 19 Xinjiekouwai Street, Beijing, 100875, PR China. Tel: +86-18910970384; E-mail: yuanchun-ren@bnu.edu.cn

**Li Yang**, MD, PhD

Department of Child & Adolescent Psychiatry, Peking University Sixth Hospital (Institute of Mental Health), National Clinical Research Center for Mental Disorders and NHC Key Laboratory of Mental Health (Peking University Sixth Hospital), 51 HuayuanBei Road, Beijing, 100191, PR China. Tel: +86 6235-0880; E-mail: yangli_pkuimh@bjmu.edu.cn

**ONLINE APPENDIX**

# Index

**Appendix A.** Additional details on search strategy

**Supplementary Table 1.** PRISMA Checklist

**Supplementary Table 2.** Other characteristics of the included studies

**Supplementary Table 3.** Pooled effect size for physical activity intervention on ADHD-related emotional problems

**Supplementary Table 4.** Pooled effect size for physical activity intervention on ADHD-related behavioral problems

**Supplementary Figure 1.** Funnel plots of eligible studies

# Appendix A. Additional details on search strategy

Search in electronic sources

- This study followed the preferred reporting items for systematic reviews and meta-analyses (1) statement (2).
- The following electronic databases were searched PubMed, EMBASE, Web of Science Core Collection.

Search syntax for each database

1. PubMed 2,866

#1 “Attention Deficit Disorder with Hyperactivity”[MESH]

#2 “Attention Deficit Disorder with Hyperactivit*” OR “attention-deficit hyperactivity disorder*” OR “attention deficit disorder*” OR “ADHD” OR adhd OR addh OR add OR “Attention Deficit Disorder* with Hyperactivity” OR “Attention Deficit Hyperactivity Disorder*” OR “Attention Deficit-Hyperactivity Disorder*” OR “attention deficit disorder hyperactivity” OR “attention deficit” OR “child attention deficit disorder” OR hyperactiv* OR “Hyperkinetic Syndrome*” OR “syndrome* hyperkinetic” OR “hyperkinetic syndrome childhood” OR “attention deficit hyperkinetic disorder” OR “hyperkinetic disorder” OR hyperkinet* OR overactive* OR “overactive child syndrome” OR inattenti* OR “attention problem*” OR “syndrome hyperkinetic” OR “hyperkinetic syndrome” OR “hyperactivity disorder” OR “hyperactive child syndrome” OR “childhood hyperkinetic syndrome”

#3 "Sports"[Mesh] OR "Exercise"[Mesh]

#4 Sport* OR Exercis* OR “Locomotor Activit*” OR “Physical* therap*” OR “physical* activit*” OR “motor activit*” OR Soccer OR Swim* OR Aquatic* OR Dive OR Diving OR Football OR Pin pang OR Ping-pong OR Ping pang OR Basketball OR Cricket OR Tennis OR Rugby OR Danc* OR Athletic* OR “Martial art*” OR Netball OR Hockey OR Gym* OR “horse rid*” OR “horseback rid*” OR Equestrian OR Baseball OR Yoga OR Badminton OR Taekwondo OR Danc* OR Judo OR Cycling

#5 #1 OR #2

#6 #3 OR #4

#7 #5 AND #6

The last search was run on June, 2021.

1. Web of Science 2,660

#1 TI = (“Attention Deficit Disorder with Hyperactivit*” OR “attention-deficit hyperactivity disorder*” OR “attention deficit disorder*” OR “ADHD” OR adhd OR addh OR add OR “Attention Deficit Disorder* with Hyperactivity” OR “Attention Deficit Hyperactivity Disorder*” OR “Attention Deficit-Hyperactivity Disorder*” OR “attention deficit disorder hyperactivity” OR “attention deficit” OR “child attention deficit disorder” OR hyperactiv* OR “Hyperkinetic Syndrome*” OR “syndrome* hyperkinetic” OR “hyperkinetic syndrome childhood” OR “attention deficit hyperkinetic disorder” OR “hyperkinetic disorder” OR hyperkinet* OR overactive* OR “overactive child syndrome” OR inattenti* OR “attention problem*” OR “syndrome hyperkinetic” OR “hyperkinetic syndrome” OR “hyperactivity disorder” OR “hyperactive child syndrome” OR “childhood hyperkinetic syndrome”)

#2 TS = (Attention Deficit Disorder with Hyperactivity)

#3 TI = (Sport* OR Exercis* OR “Locomotor Activit*” OR “Physical* therap*” OR “physical* activit*” OR “motor activit*” OR Soccer OR Swim* OR Aquatic* OR Dive OR Diving OR Football OR Pin pang OR Ping-pong OR Ping pang OR Basketball OR Cricket OR Tennis OR Rugby OR Danc* OR Athletic* OR “Martial art*” OR Netball OR Hockey OR Gym* OR “horse rid*” OR “horseback rid*” OR Equestrian OR Baseball OR Yoga OR Badminton OR Taekwondo OR Danc* OR Judo OR Cycling)

#4 TS = (Sports OR Exercise)

#5 #1 OR #2

#6 #3 OR #4

#7 #5 AND #6

The last search was run on June, 2021.

1. Cochrane Library 2,193

#1 Attention Deficit Disorder with Hyperactivit* OR attention-deficit hyperactivity disorder* OR attention deficit disorder* OR ADHD OR adhd OR addh OR add OR Attention Deficit Disorder* with Hyperactivity OR Attention Deficit Hyperactivity Disorder* OR Attention Deficit-Hyperactivity Disorder* OR attention deficit disorder hyperactivity OR attention deficit” OR child attention deficit disorder OR hyperactiv* OR Hyperkinetic Syndrome* OR syndrome* hyperkinetic OR hyperkinetic syndrome childhood OR attention deficit hyperkinetic disorder OR hyperkinetic disorder OR hyperkinet* OR overactive* OR “overactive child syndrome OR inattenti* OR attention problem* OR syndrome hyperkinetic OR hyperkinetic syndrome OR hyperactivity disorder OR hyperactive child syndrome OR childhood hyperkinetic syndrome

#2 Attention Deficit Disorder with Hyperactivity [Mesh]

#3 Sport* OR Exercis* OR “Locomotor Activit*” OR “Physical* therap*” OR “physical* activit*” OR “motor activit*” OR Soccer OR Swim* OR Aquatic* OR Dive OR Diving OR Football OR Pin pang OR Ping-pong OR Ping pang OR Basketball OR Cricket OR Tennis OR Rugby OR Danc* OR Athletic* OR “Martial art*” OR Netball OR Hockey OR Gym* OR “horse rid*” OR “horseback rid*” OR Equestrian OR Baseball OR Yoga OR Badminton OR Taekwondo OR Danc* OR Judo OR Cycling

#4 Sports [Mesh]

#5 Exercise [Mesh]

#6 #1 OR #2

#7 #3 OR #4 OR #5

#8 #6 AND #7

The last search was run on June, 2021.

1. Embase 1,574

#1 ‘attention deficit disorder with hyperactivit*’ OR ‘attention-deficit hyperactivity disorder*’ OR ‘attention deficit disorder*’ OR ‘adhd’ OR adhd OR addh OR add OR ‘attention deficit disorder* with hyperactivity’ OR ‘attention deficit hyperactivity disorder*’ OR ‘attention deficit-hyperactivity disorder*’ OR ‘attention deficit disorder hyperactivity’ OR ‘attention deficit’ OR ‘child attention deficit disorder’ OR hyperactiv* OR ‘hyperkinetic syndrome*’ OR ‘syndrome* hyperkinetic’ OR ‘hyperkinetic syndrome childhood’ OR ‘attention deficit hyperkinetic disorder’ OR ‘hyperkinetic disorder’ OR hyperkinet* OR overactive* OR ‘overactive child syndrome’ OR inattenti* OR ‘attention problem*’ OR ‘syndrome hyperkinetic’ OR ‘hyperkinetic syndrome’ OR ‘hyperactivity disorder’ OR ‘hyperactive child syndrome’ OR ‘childhood hyperkinetic syndrome’

#2 ‘attention deficit disorder’/exp

#3 sport* OR exercis* OR ‘locomotor activit*’ OR ‘physical* therap*’ OR ‘physical* activit*’ OR ‘motor activit*’ OR soccer OR swim* OR aquatic* OR dive OR diving OR football OR ‘pin pang’ OR ‘ping pong’ OR ‘ping pang’ OR basketball OR cricket OR tennis OR rugby OR athletic* OR ‘martial art*’ OR netball OR hockey OR gym* OR ‘horse rid*’ OR ‘horseback rid*’ OR equestrian OR baseball OR yoga OR badminton OR taekwondo OR danc* OR judo OR cycling

#4 ‘sport’/exp

#5 ‘exercise’/exp

#6 #1 OR #2

#7 #3 OR #4 OR #5

#8 #6 AND #7

The last search was run on June, 2021.

# Supplementary Table 1. PRISMA Checklist

**Title of submitted paper and corresponding author**:

| **#** | **Item** | **Guidance** | **On page #** |
| --- | --- | --- | --- |
| **Title** | | | |
| 1 | Title | Identify the report as a systematic review, or systematic review and meta-analysis, as appropriate. | 1 |
| **Abstract** | | | |
| 2 | Structured summary | Provide a structured summary including, as applicable:   - Background; - Objectives; - Data sources; - Study eligibility criteria, participants, and interventions; - Study appraisal and synthesis methods; - Results; - Limitations; conclusions and implications of key findings; - Systematic review registration number. | 1-2 |
| **Introduction** | | | |
| 3 | Rationale | Describe the rationale for the review in the context of what is already known. | 2-3 |
| 4 | Objectives | Provide an explicit Population-Intervention-Comparator-Outcome-Study Design (PICOS) or Population-Exposure-Comparator-Outcome-Study Design (PECOS) statement as appropriate, detailing the following in relation to the research questions being asked:   - Participants - Interventions / Exposures (as appropriate) - Comparisons - Outcomes - Study design | 3 |
| **Methods** | | | |
| 5 | Protocol and registration | Indicate if a review protocol exists, if and where it can be accessed (e.g. web address), and registration information including registration number (if available). | NR |
| 6 | Eligibility criteria | Specify study characteristics (e.g. PICOS/PECOS, length of exposure) and report characteristics (e.g. years considered, language, publication status) used as criteria for eligibility, giving rationale. | 3-4 |
| 7 | Information sources | Describe all information sources (e.g. databases with dates of coverage, contact with study authors to identify additional studies) in the search, and date last searched. | 3 |
| 8 | Search | Present full electronic search strategy for at least one database, including any limits used, such that it could be repeated. | 3, Appendix A |
| 9 | Study selection | State the process for selecting studies (i.e., screening, eligibility, included in systematic review, and, if applicable, included in the meta-analysis). | 3-4 |
| 10 | Data collection process | Describe method of data extraction from reports (e.g., piloted forms, independently, in duplicate) and any processes for obtaining and confirming data from investigators. | 4 |
| 11 | Data items | List and define all variables for which data were sought (e.g., PICOS/PECOS, funding sources) and any assumptions and simplifications made. | 4 |
| 12 | Risk of bias in individual studies | Describe methods used for assessing risk of bias of individual studies (including specification of whether this was done at the study or outcome level), and how this information is to be used in any data synthesis. | 4 |
| 13 | Summary measures | State the principal summary measures (e.g., risk ratio, difference in means). | 4 |
| 14 | Synthesis of results | Describe the methods of handling data and combining results of studies, if done, including measures of consistency (e.g., I^2^) for each meta-analysis. | 4 |
| 15 | Risk of bias across studies | Specify any assessment of risk of bias that may affect the cumulative evidence (e.g., publication bias, selective reporting within studies). | 4 |
| 16 | Additional analyses | Describe methods of additional analyses (e.g., sensitivity or subgroup analyses, meta-regression), if done, indicating which were pre-specified. | 4-5 |
| **Results** | | | |
| 17 | Study selection | Give numbers of studies screened, assessed for eligibility, and included in the review, with reasons for exclusions at each stage, illustrated with a PRISMA flow diagram. | 5, Figure 1 |
| 18 | Study characteristics | For each study, present in a summary table the characteristics for which data were extracted (e.g., study size, PICOS/PECOS, follow-up period) and provide the citations. | 5, Table 1, and Supplementary Table 2 |
| 19 | Risk of bias within studies | Present data on risk of bias of each study and, if available, any outcome level assessment (see item 12). | 6, Supplementary Figure 1 |
| 20 | Results of individual studies | For all outcomes considered (benefits or harms), present, for each study: (a) simple summary data for each intervention group (b) effect estimates and confidence intervals, ideally with a forest plot (unless such a plot would be misleading) | Figure 2 and 3 |
| 21 | Synthesis of results | Present results of each meta-analysis done, including confidence intervals and measures of consistency. | 5-6, Table 2 and 3 |
| 22 | Risk of bias across studies | Present results of any assessment of risk of bias across studies (see Item 15). | Table 2 and 3 |
| 23 | Additional analysis | Give results of additional analyses, if done (e.g., sensitivity or subgroup analyses, meta-regression [see Item 16]). | 5-6, Table 2 and 3, Supplementary Table 3 and 4 |
| **Discussion** | | | |
| 24 | Summary of evidence | Summarize the main findings including the strength of evidence for each main outcome; consider their relevance to key groups (e.g., researchers, users, and policy makers). | 6 |
| 25 | Limitations | Discuss limitations at study and outcome level (e.g., risk of bias), and at review-level (e.g., incomplete retrieval of identified research, reporting bias). | 8 |
| 26 | Conclusions | Provide a general interpretation of the results in the context of other evidence, and implications for future research. | 8 |
| 27 | Funding | Describe sources of funding for the systematic review and other support (e.g., supply of data); role of funders for the systematic review. | 8 |

# Supplementary Table 2. Other characteristics of the included studies

| **Study** | **Race** | **Physical activity** | **Type** | **Measurement** | **Version** | **Outcomes** | | | |
| --- | --- | --- | --- | --- | --- | --- | --- | --- | --- |
|  |  |  |  |  |  | Inattention | Hyperactivity/impulsivity | Emotional problems | Behavioral problems |
| Jensen et al. (2004, Australia) (3) | 18 Caucasian and 1 Chinese. | Yoga | Closed | Conners’ Teacher Rating Scale–Revised: Long (CTRS– R: L) | Teacher | DSM-IV Inattentive | DSM-IV Hyperactive/impulsive | Anxious/Shy | Oppositional |
|  |  |  |  | Conners’ Parent Rating Scale–Revised: Long (CPRS–R: L) | Parent | DSM-IV Inattentive | DSM-IV Hyperactive/impulsive | Anxious/Shy | Oppositional |
| Verret et al. (2012, Canada) (4) | NR | Basketball, soccer, exercise stations, and tag and ball games | Open | Child Behavior Checklist (CBCL) | Parent | Attention problems |  | Anxiety-depression  Withdrawn-depression | Rule-breaking behaviors  Aggressive behaviors |
| So et al. (2017, Republic of Korea) (5) | NR | Horseback riding | Open | The ADHD Rating Scale | Parent/teacher | Inattention |  |  |  |
|  |  |  |  | Revised Children’s Manifest Anxiety Scale (RCMAS) | Self-report |  |  | Anxiety |  |
|  |  |  |  | The Center for Epidemiological Studies Depression Scale (CES-D) | Self-report |  |  | Depression |  |
| Kallweit et al. (2019, Germany) (6) | NR | Bicycle ergometer | Closed | Questionnaire constructed based on DSM-IV criteria for ADHD | Self-report | Inattention | Hyperactivity |  |  |
| Converse et al. (2020, America) (7) | White 86 %. | Tai Chi | Closed | Conners Adult ADHD Rating Scales self-report long version (CAARS-S: L) | Self-report | DSM IV Inattentive | DSM-IV Hyperactive/impulsive |  |  |
| Kang et al. (2011, Korea) (8) | NR | Aerobic exercise, goal-directed exercise, jump throwing tennis balls, jump roping | Closed | Korean ADHD Rating Scale parent and teacher version (K-ARS-PT) | Parent/teacher | Inattention | Hyperactivity |  |  |
| Hoza et al. (2015, America) (9) | Caucasian 68.3%, Mixed race 14.4%, African American 7.9 %, Asian 2.0 %, and other races 7.4 %. | Games | Open | The ADHD-IV Rating Scale | Parent | Inattention symptoms | Hyperactive/impulsive symptoms |  |  |
|  |  |  |  | Revised version of the Oppositional/Defiant subscale of the Pittsburgh Modified Conners Parent and Teacher Rating Scale | Parent |  |  | Moodiness | Oppositional symptoms |
|  |  |  |  | The ADHD-IV Rating Scale | Teacher | Inattention symptoms | Hyperactive/impulsive symptoms |  |  |
|  |  |  |  | Revised version of the Oppositional/Defiant subscale of the Pittsburgh Modified Conners Parent and Teacher Rating Scale | Teacher |  |  | Moodiness | Oppositional symptoms |
| Zhang et al. (2015, China) (10) | NR | Games | Open | Beck depression inventory (BDI) | Parent |  |  | Depression |  |
| Pan et al. (2016, China) (11) | NR | Racket-sport | Open | Chinese version of the Child Behavior Checklist (CBCL) | Parent | Attention problems |  | Anxiety-depression  Withdrawn-depression | Rule-breaking behaviors  Aggressive behaviors |
| Bustamante et al. (2016, America) (12) | Control group:  African American 94%, Latino 6%.  Treatment group:  African American 100%. | Games | Open | Disruptive behavior disorders (DBD) Rating Scale | Parent | Inattention severity | Hyperactivity severity |  | Oppositional defiant disorder (ODD) severity |
| García-Gómez et al. (2016, Spain) (13) | NR | Equestrian therapies | Open | Spanish version of teacher-rating Behavioral Assessment System for Children (BASC-T) | Teacher | Attention problems | Hyperactivity | Depression  Anxiety  Withdrawal | Conduct problems |
| Geladé et al. (2017, Netherlands) (14) | NR | Semi‑active control condition | NR | Strengths and Weaknesses of ADHD symptoms and Normal Behavior Scale (SWAN) | Parent | Inattention | Hyperactivity/impulsivity |  |  |
|  |  |  |  | Strengths and Weaknesses of ADHD symptoms and Normal Behavior Scale (SWAN) | Teacher | Inattention | Hyperactivity/impulsivity |  |  |
| Oh et al. (2018, Korea) (15) | NR | Hippotherapy | Open | The ADHD Rating Scale (ARS) | NR | Inattention | Hyperactivity/impulsivity |  |  |
|  |  |  |  | The Child Behavior Checklist  (CBCL) | Parent |  |  | Withdrawn/depressed  Anxious/depressed | Rule-breaking behavior  Aggressive behavior |
| Silva et al. (2019, Brasilia) (16) | NR | Swimming | Closed | The Child Depression Inventory (CDI) | NR |  |  | Depression |  |
|  |  |  |  | Adapted Beck Anxiety Inventory (BAI) | NR |  |  | Anxiety |  |
| Hernandez-Reif et al. (2000, America) (17) | White Anglo 70%, Hispanic 15%, and African American 15%. | Tai Chi | Closed | Conner’s Teacher Rating Scale– Revised (CTRS-R) | Teacher |  | Hyperactive | Anxiety  Emotion | Conduct |
| Lufi et al. (2011, Israel) (18) | NR | Obstacle races, relay races, and calisthenics | Open | Youth Self-Report (YSR) | Self-report | Attention |  | Withdrawn  Anxiety | Aggression  Delinquency |
|  |  |  |  | The Child Behavior Checklist (CBCL) | Parent | Attention |  | Withdrawn  Anxiety | Aggression  Delinquency |
|  |  |  |  | The Conners’ Abbreviated Symptom Questionnaire-Parents (ASQ-P) | Parent |  | Hyperactivity |  |  |
| Smith et al. (2013, America) (19) | 12 White and 2 Black. | Games | Open | Pittsburgh Modified Conners Teacher Rating Scale (PMCTRS) | Teacher |  | Hyperactivity |  | Oppositional/defiant |
| Cuypers et al. (2011, Norway) (20) | NR | Horseback Riding | Open | The Strength and Difficulties Questionnaire (SDQ) | Self-report |  |  | Emotional symptoms | Conduction problems |
| Fritz et al. (2015, America) (21) | NR | Cycling | Closed | Profile of Mood States–Brief Form (POMS-BF) | Self-report |  |  | Tension  Anger  Depression |  |
| Jang et al. (2015, Korea) (22) | NR | Equine-assisted activities | Open | ADHD-Rating Scale (ARS-I) | NR | Inattention | Hyperactivity/impulsivity |  |  |
|  |  |  |  | The Korea-Child Behavior Checklist (K-CBCL) | Parent |  |  | Withdrawn  Anxiety/depression | Delinquent behavior  Aggressive behavior |
| Schoenfelder et al. (2017, America) (23) | Caucasian 80%, Asian American 10%, and Multi-racial 10%. | Walk or run | Closed | Vanderbilt ADHD Diagnostic Parent Rating Scale (VADPRS) | Parent | ADHD-inattentive symptoms | ADHD-hyperactive/impulsive symptoms |  |  |
|  |  |  |  | Adjusted Vanderbilt ADHD Diagnostic Parent Rating Scale | Self-report | ADHD-inattentive symptoms | ADHD-hyperactive/impulsive symptoms |  |  |
|  |  |  |  | Positive and Negative Affect Schedule for Children (PANAS-C) | Self-report |  |  | Negative mood |  |
| Shema-Shiratzky et al. (2019, Israel) (24) | NR | Virtual reality training | Open | The Conners’ Parent Rating Scale-Revised (CPRS-R) | Parent | DSM-IV Inattentive | DSM-IV Hyperactive-impulsive | Anxious-shy behavior | Oppositional behavior |
| Siu et al. (2020, China) (25) | NR | Rugby | Open | Chinese version of the Strengths and Weaknesses of ADHD-symptoms and Normal behavior (SWAN) | Parent | Inattention | Hyperactivity/impulsivity |  |  |

**Abbreviations:** NR, not report.

# Supplementary Table 3. Pooled effect size for physical activity intervention on ADHD-related emotional problems

|  | **Before-after studies** | | | | | | |  | **Two-group control studies** | | | | | | |
| --- | --- | --- | --- | --- | --- | --- | --- | --- | --- | --- | --- | --- | --- | --- | --- |
|  | **No.** | **Effect size** | | |  | **Heterogeneity** | |  | **No.** | **Effect size** | | |  | **Heterogeneity** | |
|  |  | **SMD** | **95% CI** | **P for Z** |  | **I^2^** | **P** |  |  | **SMD** | **95% CI** | **P for Z** |  | **I^2^** | **P** |
| **Overall intervention effect** | 14 | 0.416 | 0.283, 0.549 | <0.001 |  | 28.5% | 0.151 |  | 6 | 0.469 | -0.156, 1.094 | 0.141 |  | 85.4% | <0.001 |
| **ADHD diagnostic status** |  |  |  |  |  |  |  |  |  |  |  |  |  |  |  |
| Yes | 12 | 0.394 | 0.194, 0.595 | <0.001 |  | 15.9% | 0.288 |  | 5 | 0.684 | -0.173, 1.542 | 0.118 |  | 84.9% | <0.001 |
| No | 2 | 0.469 | 0.026, 0.912 | 0.038 |  | 79.0% | 0.029 |  | 1 | -0.159 | -0.446, 0.128 | 0.277 |  | - | - |
| **Study population** |  |  |  |  |  |  |  |  |  |  |  |  |  |  |  |
| Children | 13 | 0.357 | 0.188, 0.525 | <0.001 |  | 12.6% | 0.319 |  | 6 | 0.469 | -0.156, 1.094 | 0.141 |  | 85.4% | <0.001 |
| Adults | 1 | 0.697 | 0.404, 0.990 | <0.001 |  | - | - |  | - | - | - | - |  | - | - |
| **Motor skills** |  |  |  |  |  |  |  |  |  |  |  |  |  |  |  |
| Closed | 5 | 0.625 | 0.364, 0.885 | <0.001 |  | 5.4% | 0.376 |  | 1 | 0.040 | -0.607, 0.688 | 0.904 |  | - | - |
| Open | 9 | 0.421 | 0.248, 0.594 | <0.001 |  | 14.7% | 0.311 |  | 5 | 0.591 | -0.166, 1.347 | 0.126 |  | 88.3% | <0.001 |
| **Intensity of physical activity** |  |  |  |  |  |  |  |  |  |  |  |  |  |  |  |
| Moderate | 4 | 0.709 | 0.303, 1.116 | 0.001 |  | 55.8% | 0.079 |  | 2 | 1.449 | -0.767, 3.666 | 0.200 |  | 94.4% | <0.001 |
| Moderate-to-vigorous | 1 | 0.245 | -0.036, 0.526 | 0.088 |  | - | - |  | 2 | 0.390 | -1.085, 1.865 | 0.605 |  | 67.7% | 0.078 |
| **Frequency of physical activity** |  |  |  |  |  |  |  |  |  |  |  |  |  |  |  |
| <3 times per week | 9 | 0.342 | 0.146, 0.537 | 0.001 |  | 0.0% | 0.440 |  | 2 | 0.033 | -0.761, 0.827 | 0.935 |  | 71.6% | 0.060 |
| ≥3 times per week | 2 | 0.685 | -0.304, 1.675 | 0.175 |  | 82.4% | 0.017 |  | 3 | 1.253 | -0.804, 3.310 | 0.233 |  | 93.4% | <0.001 |
| **Medication** |  |  |  |  |  |  |  |  |  |  |  |  |  |  |  |
| Yes | 1 | 0.260 | -0.580, 1.100 | 0.544 |  | - | - |  | - | - | - | - | - | - | - |
| No | 5 | 0.268 | 0.021, 0.516 | 0.033 |  | 0% | 0.989 |  | - | - | - | - | - | - | - |

**Abbreviations:** 95% CI, 95% confidence interval; SMD, standardized mean difference.

# Supplementary Table 4. Pooled effect size for physical activity intervention on ADHD-related behavioral problems

|  | **Before-after studies** | | | | | | |  | **Two-group control studies** | | | | | | |
| --- | --- | --- | --- | --- | --- | --- | --- | --- | --- | --- | --- | --- | --- | --- | --- |
|  | **No.** | **Effect size** | | |  | **Heterogeneity** | |  | **No.** | **Effect size** | | |  | **Heterogeneity** | |
|  |  | **SMD** | **95% CI** | **P for Z** |  | **I^2^** | **P** |  |  | **SMD** | **95% CI** | **P for Z** |  | **I^2^** | **P** |
| **Overall intervention effect** | 12 | 0.347 | 0.202, 0.492 | <0.001 |  | 0.0% | 0.966 |  | 6 | 0.100 | -0.279, 0.479 | 0.605 |  | 66.2% | 0.011 |
| **ADHD diagnostic status** |  |  |  |  |  |  |  |  |  |  |  |  |  |  |  |
| Yes | 10 | 0.346 | 0.171, 0.521 | <0.001 |  | 0.0% | 0.992 |  | 4 | 0.382 | 0.108, 0.656 | 0.006 |  | 3.5% | 0.375 |
| No | 2 | 0.455 | -0.057, 0.966 | 0.082 |  | 53.7% | 0.142 |  | 2 | -0.256 | -0.522, 0.009 | 0.058 |  | 0.0% | 0.336 |
| **Study population** |  |  |  |  |  |  |  |  |  |  |  |  |  |  |  |
| Children | 12 | 0.347 | 0.202, 0.492 | <0.001 |  | 0.0% | 0.966 |  | 6 | 0.100 | -0.279, 0.479 | 0.605 |  | 66.2% | 0.011 |
| Adults | - | - | - | - |  | - | - |  | - | - | - | - |  | - | - |
| **Motor skills** |  |  |  |  |  |  |  |  |  |  |  |  |  |  |  |
| Closed | 2 | 0.613 | -0.065, 1.291 | 0.076 |  | 0.0% | 0.635 |  | 1 | 0.119 | -0.535, 0.773 | 0.721 |  | - | - |
| Open | 10 | 0.334 | 0.186, 0.483 | <0.001 |  | 0.0% | 0.952 |  | 5 | 0.098 | -0.351, 0.548 | 0.668 |  | 72.9% | 0.005 |
| **Intensity of physical activity** |  |  |  |  |  |  |  |  |  |  |  |  |  |  |  |
| Moderate | 1 | 0.362 | 0.010, 0.713 | 0.044 |  | - | - |  | 1 | 0.365 | 0.013, 0.716 | 0.042 |  | - | - |
| Moderate-to-vigorous | 3 | 0.381 | 0.099, 0.663 | 0.008 |  | 9.8% | 0.330 |  | 3 | 0.017 | -0.695, 0.728 | 0.964 |  | 80.1% | 0.007 |
| **Frequency of physical activity** |  |  |  |  |  |  |  |  |  |  |  |  |  |  |  |
| <3 times per week | 7 | 0.337 | 0.150, 0.524 | <0.001 |  | 0.0% | 0.931 |  | 2 | 0.333 | -0.002, 0.668 | 0.051 |  | 0.0% | 0.559 |
| ≥3 times per week | 4 | 0.362 | 0.129, 0.595 | 0.002 |  | 0.0% | 0.528 |  | 3 | 0.017 | -0.695, 0.728 | 0.964 |  | 80.1% | 0.007 |
| **Medication** |  |  |  |  |  |  |  |  |  |  |  |  |  |  |  |
| Yes | 1 | 0.579 | -0.692, 1.851 | 0.372 |  | - | - |  | - | - | - | - | - | - | - |
| No | 6 | 0.332 | 0.086, 0.578 | 0.008 |  | 0% | 0.973 |  | - | - | - | - | - | - | - |

**Abbreviations:** 95% CI, 95% confidence interval; SMD, standardized mean difference.

# Supplementary Figure 1. Funnel plots of eligible studies


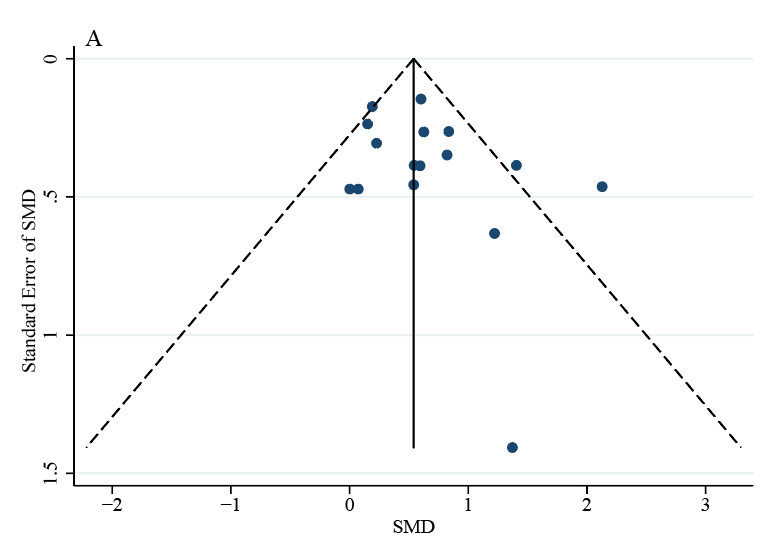

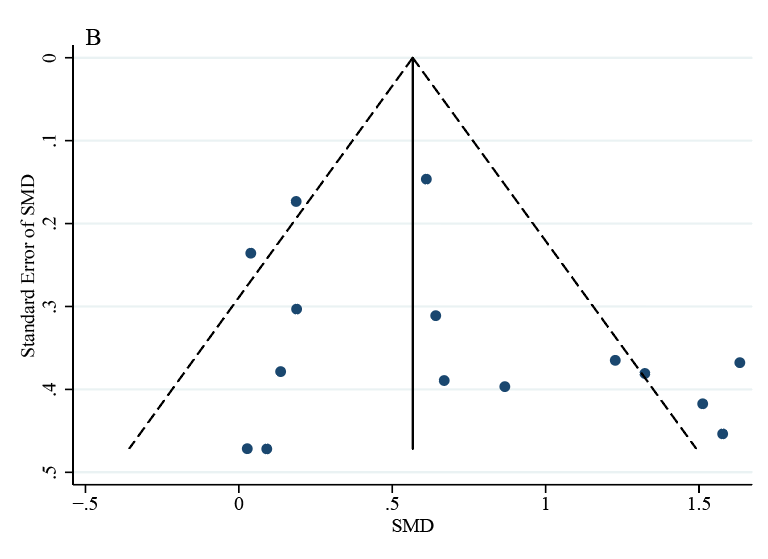

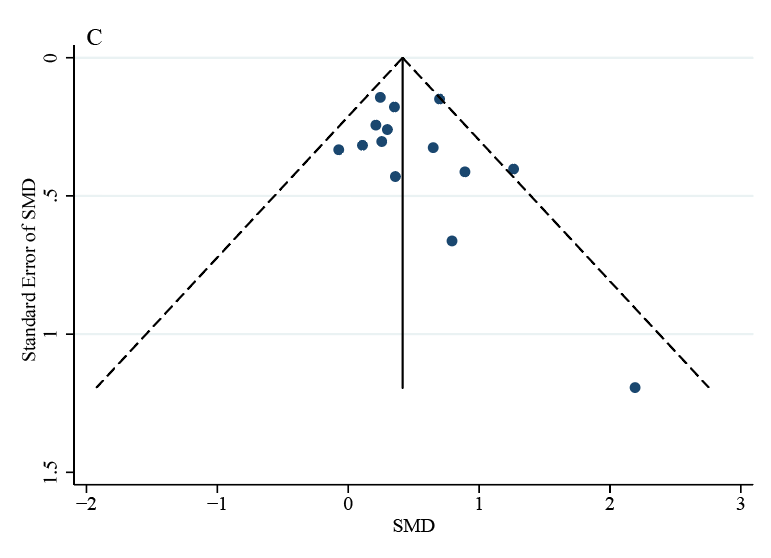

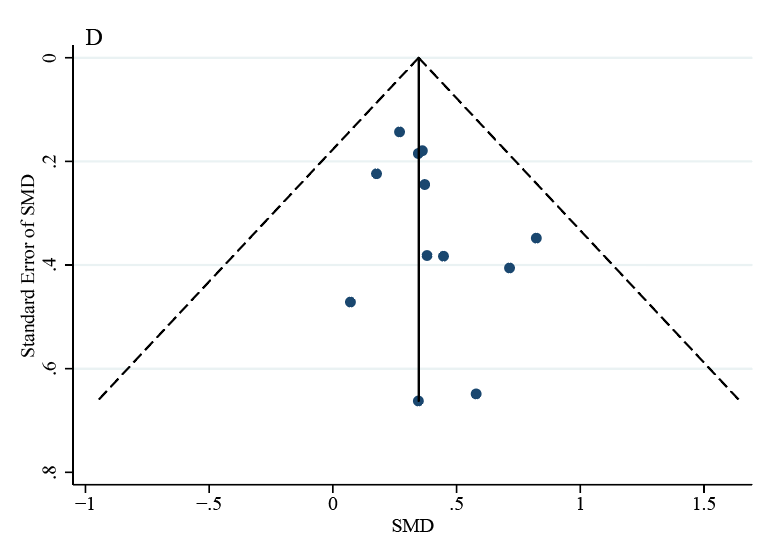

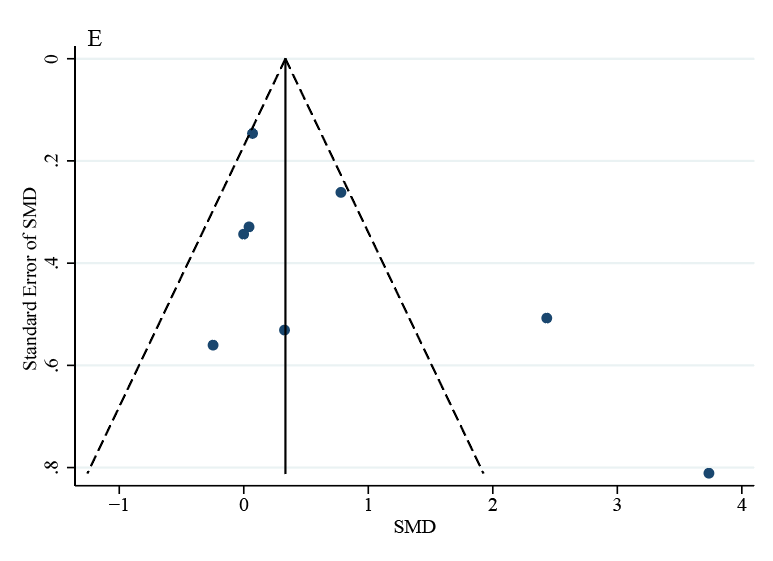

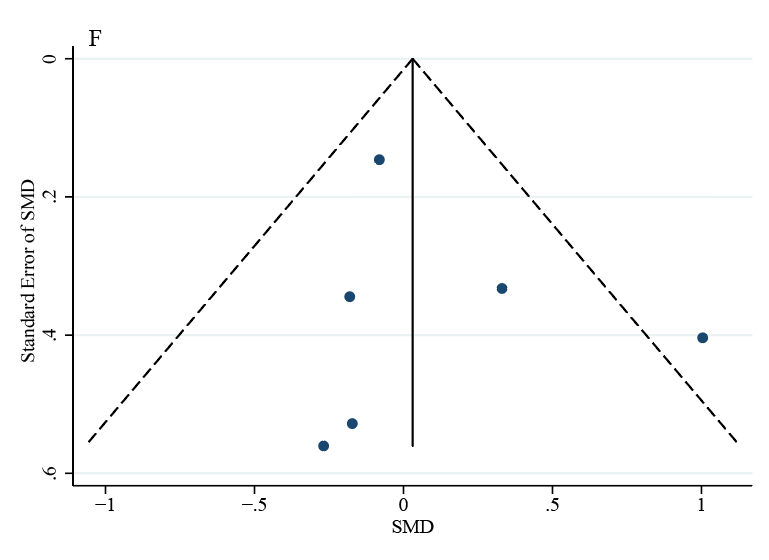

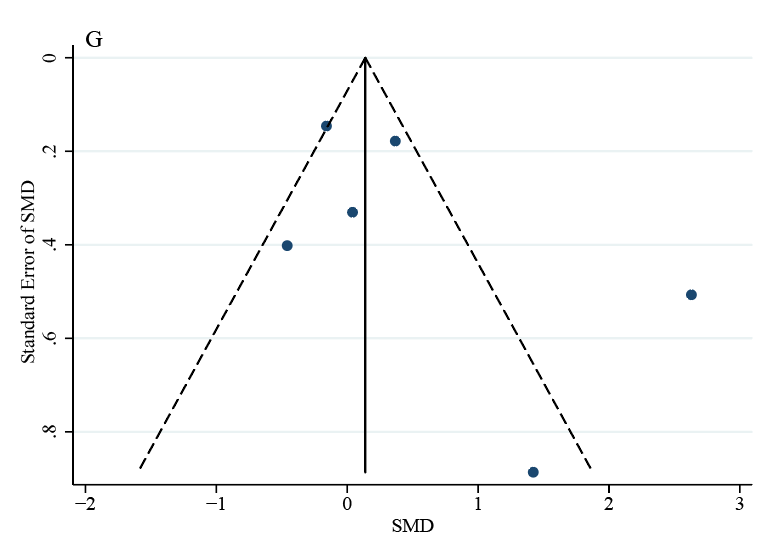

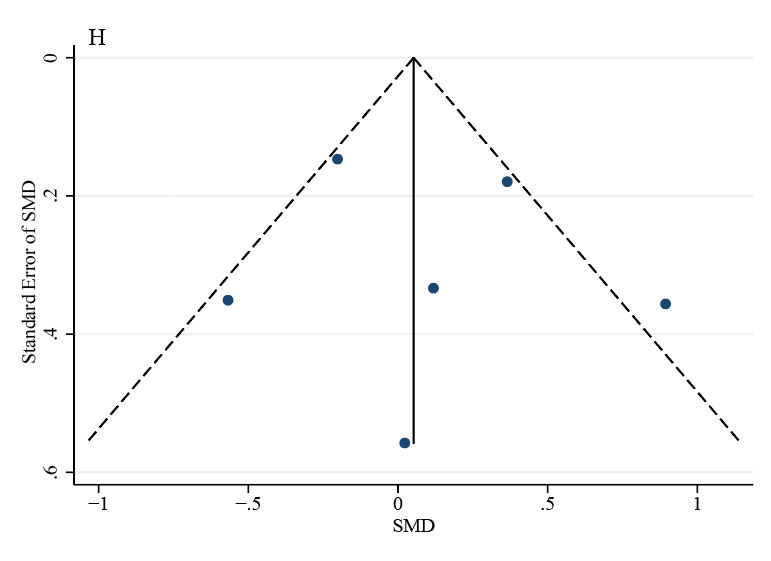


**Notes:** Before-after study: A) attention problems; B) hyperactive/impulsive problems; C) emotional problems; D) behavioral problems, Two-group control study: E) attention problems; F) hyperactive/impulsive problems; G) emotional problems; H) behavioral problems.

**Abbreviations:** SMD, standardized mean difference.

# Reference:

1. Moher D, Liberati A, Tetzlaff J, et al. Preferred Reporting Items for Systematic Reviews and Meta-Analyses: The PRISMA Statement. *J Clin Epidemiol* 2009;62(10):1006-12.

2. Moher D, Liberati A, Tetzlaff J, et al. Preferred reporting items for systematic reviews and meta-analyses: the PRISMA statement. *PLoS Med* 2009;6(7):e1000097.

3. Jensen PS, Kenny DT. The effects of yoga on the attention and behavior of boys with Attention-Deficit/ hyperactivity Disorder (ADHD). *J Atten Disord* 2004;7(4):205-16.

4. Verret C, Guay M-C, Berthiaume C, et al. A Physical Activity Program Improves Behavior and Cognitive Functions in Children With ADHD: An Exploratory Study. *Journal of Attention Disorders* 2012;16(1):71-80.

5. So W-Y, Lee S-Y, Park Y, et al. Effects of 4 Weeks of Horseback Riding on Anxiety, Depression, and Self-Esteem in Children with Attention Deficit Hyperactivity Disorder. *Journal of Men's Health* 2017;13(2).

6. Kallweit C, Paucke M, Strauss M, et al. Adult ADHD: Influence of Physical Activation, Stimulation, and Reward on Cognitive Performance and Symptoms. *Journal of Attention Disorders* 2021;25(6):809-19.

7. Converse AK, Barrett BP, Chewning BA, et al. Tai Chi training for attention deficit hyperactivity disorder: A feasibility trial in college students. *Complement Ther Med* 2020;53:102538.

8. Kang KD, Choi JW, Kang SG, et al. Sports Therapy for Attention, Cognitions and Sociality. *Int J Sports Med* 2011;32(12):953-9.

9. Hoza B, Smith AL, Shoulberg EK, et al. A randomized trial examining the effects of aerobic physical activity on attention-deficit/hyperactivity disorder symptoms in young children. *J Abnorm Child Psychol* 2015;43(4):655-67.

10. Zhang LL, Ji WL, Fan YZ, et al. Effect of Physical Exercise on Cognition and Emotion in Children with ADHD. *3rd Aasri Conference on Computational Intelligence and Bioinformatics (Cib 2015)* 2015:138-41.

11. Pan CY, Chu CH, Tsai CL, et al. A racket-sport intervention improves behavioral and cognitive performance in children with attention-deficit/hyperactivity disorder. *Research in Developmental Disabilities* 2016;57:1-10.

12. Bustamante EE, Davis CL, Frazier SL, et al. Randomized Controlled Trial of Exercise for ADHD and Disruptive Behavior Disorders. *Med Sci Sports Exerc* 2016;48(7):1397-407.

13. Garcia-Gomez A, Rodriguez-Jimenez M, Guerrero-Barona E, et al. Benefits of an experimental program of equestrian therapy for children with ADHD. *Res Dev Disabil* 2016;59:176-85.

14. Gelade K, Janssen TWP, Bink M, et al. A 6-month follow-up of an RCT on behavioral and neurocognitive effects of neurofeedback in children with ADHD. *Eur Child Adolesc Psychiatry* 2018;27(5):581-93.

15. Oh Y, Joung YS, Jang B, et al. Efficacy of Hippotherapy Versus Pharmacotherapy in Attention-Deficit/Hyperactivity Disorder: A Randomized Clinical Trial. *J Altern Complement Med* 2018;24(5):463-71.

16. Silva LAD, Doyenart R, Henrique Salvan P, et al. Swimming training improves mental health parameters, cognition and motor coordination in children with Attention Deficit Hyperactivity Disorder. *Int J Environ Health Res* 2020;30(5):584-92.

17. Hernandez-Reif M, Field TM, Thimas E. Attention deficit hyperactivity disorder: Benefits from Tai Chi. *Journal of Bodywork and Movement Therapies* 2001;5(2):120-3.

18. Lufi D, Parish-Plass J. Sport-Based Group Therapy Program for Boys with ADHD or with Other Behavioral Disorders. *Child Fam Behav Ther* 2011;33(3):217-30.

19. Smith AL, Hoza B, Linnea K, et al. Pilot physical activity intervention reduces severity of ADHD symptoms in young children. *J Atten Disord* 2013;17(1):70-82.

20. Cuypers K, De Ridder K, Strandheim A. The effect of therapeutic horseback riding on 5 children with attention deficit hyperactivity disorder: a pilot study. *J Altern Complement Med* 2011;17(10):901-8.

21. Fritz KM, O'Connor PJ. Acute Exercise Improves Mood and Motivation in Young Men with ADHD Symptoms. *Med Sci Sports Exerc* 2016;48(6):1153-60.

22. Jang B, Song J, Kim J, et al. Equine-Assisted Activities and Therapy for Treating Children with Attention-Deficit/Hyperactivity Disorder. *J Altern Complement Med* 2015;21(9):546-53.

23. Schoenfelder E, Moreno M, Wilner M, et al. Piloting a mobile health intervention to increase physical activity for adolescents with ADHD. *Prev Med Rep* 2017;6:210-3.

24. Shema-Shiratzky S, Brozgol M, Cornejo-Thumm P, et al. Virtual reality training to enhance behavior and cognitive function among children with attention-deficit/hyperactivity disorder: brief report. *Dev Neurorehabil* 2019;22(6):431-6.

25. Siu AF, Lo JW. Promising effect of a family rugby programme for children with ADHD: Promoting parent-child relationship and perceptual change on child's behaviors. *Complement Ther Clin Pract* 2020;39:101135.
